# Prominent crista terminalis mimicking a right atrial mass: case report

**DOI:** 10.1186/1476-7120-8-47

**Published:** 2010-10-19

**Authors:** Alessandro Salustri, Sherif Bakir, Amer Sana, Peter Lange, Wael Abdulrahman Al Mahmeed

**Affiliations:** 1 Institute of Cardiac Sciences, Sheikh Khalifa Medical City, Abu Dhabi, United Arab Emirates; 2 Radiology Department, Sheikh Khalifa Medical City, Abu Dhabi, United Arab Emirates

## Abstract

The crista terminalis is a normal anatomical structure within the right atrium that is not normally visualised in the standard views obtained while performing a transthoracic echocardiogram. In this case report, transthoracic echocardiography suggested the presence of a right atrial mass in a patient with end stage renal disease. However, subsequent transesophageal echocardiography revealed that the right atrial mass was actually a thick muscular bridge in the right atrium consistent with a prominent crista terminalis. An understanding of the anatomy and the echocardiographic appearance of a prominent crista terminalis will minimize the misdiagnosis of this structure avoiding unnecessary expensive additional tests.

## Background

The crista terminalis is a well-defined fibromuscular ridge formed by the junction of the sinus venosus and primitive right atrium that extends along the posterolateral aspect of the right atrial wall. Occasionally, this structure can be prominent, thus mimicking right atrial mass-like tumour, thrombus, or vegetation [1-6]. We report a case of a prominent crista terminalis recognized and properly diagnosed by echocardiography.

## Case presentation

**Figure 1:**
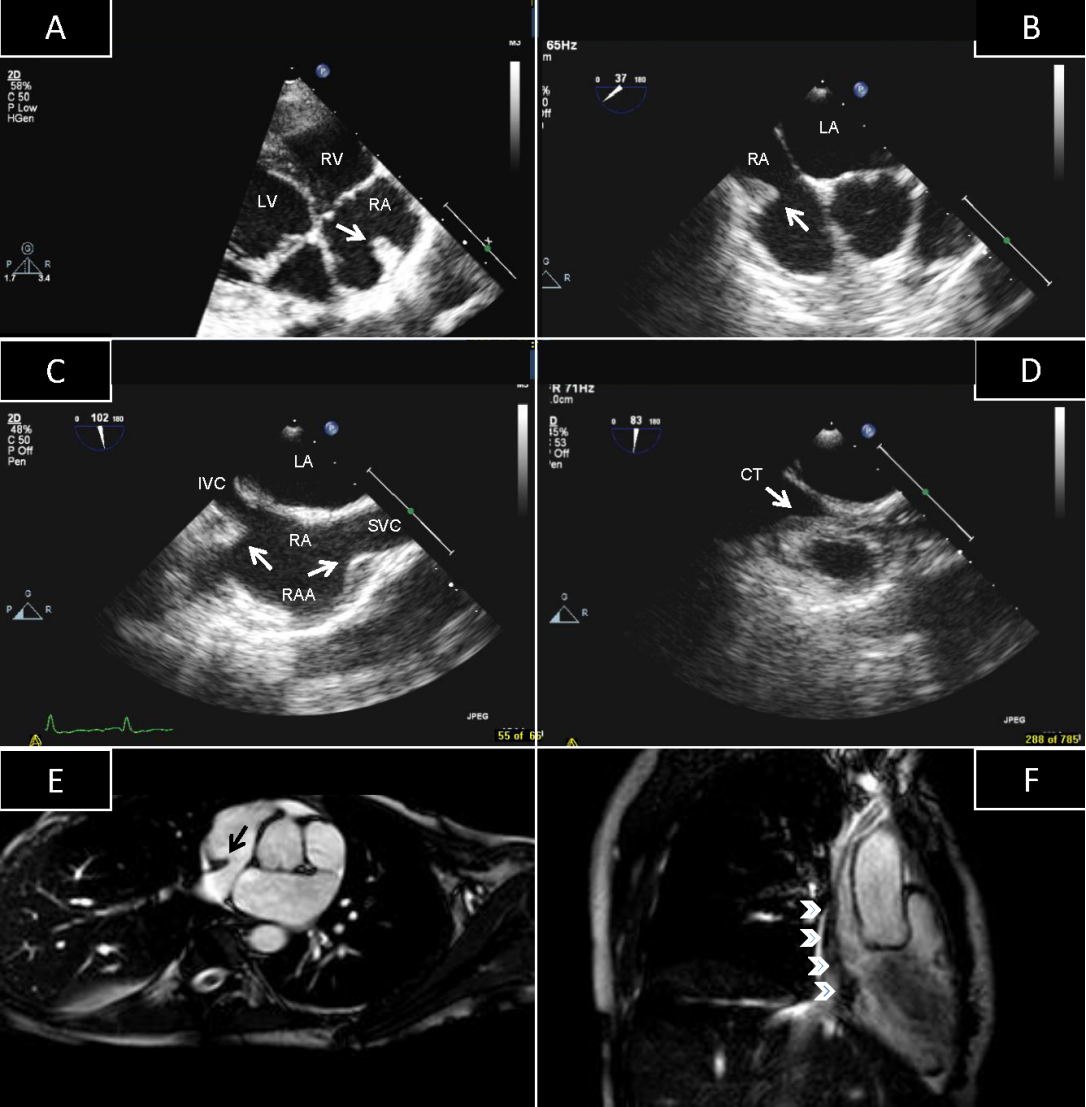
**Synoptic representation of prominent crista terminalis**. Panel A: TTE, 4-chamber view; Panel B: TEE, short-axis view at the level of the aortic valve; Panel C: TEE, bicaval view; Panel D: TEE, bicaval view, posterior side; Panel E: MRI, axial view; Panel F: MRI, cranio-caudal view. Detailed explanation in the text. Abbreviations: CT = crista terminalis; IVC = inferior vena cava; LA = left atrium; LV = left ventricle; RA = right atrium; RAA = right atrial appendage; RV = right ventricle; SVC = superior vena cava.

A 26-year-old lady with end stage renal disease was referred for transthoracic echocardiogram (TTE) as part of the departmental protocol before renal transplant. There was no history of fever, chest pain, dyspnea on exertion or palpitations. During the TTE an echogenic structure was noted in the right atrium mimicking a thrombus or a tumor (Figure 1, Panel A) (See additional files 1 and 2). Because of this finding, we performed a transesophageal echocardiogram (TEE) that showed the right atrial mass was actually a prominent crista terminalis. In the short axis at the level of the aortic valve, the superior part of the crista terminalis was most prominent (Figure 1, Panel B) (See additional file 3). Longitudinal (bicaval) views allowed clear delineation of the superior and inferior parts of this structure (Figure 1, Panel C). Careful interrogation of the region with minimal clockwise rotation of the probe clearly delineated the junction between the posterior portion of the atrium and the right atrial auricle. At this site, a well defined ridge was visualized extending from the opening of the superior vena cava to the lateral side of the entrance of the inferior vena cava (Figure 1, Panel D) (See additional file 4). Although TEE provided a comprehensive assessment of this structure, a cardiac MRI was requested by the surgical team in view of major renal surgery which confirmed the echocardiographic diagnosis. Axial MR images showed a smooth muscular ridge identified as a prominent crista terminalis on the posterolateral wall of the right atrium (Figure 1, Panel E, arrow) extending between the openings of the superior and inferior vena cava in a craniocaudal direction (Figure 1, Panel F, arrowheads). The patient underwent renal transplant and the recovery was uneventful.

## Practical Clinical Implications

The crista terminalis is originated from regression of the septum spurium as the sinus venosus is incorporated into the right atrial wall. The regression of the crista terminalis shows wide variations and thus the thickness of the crista terminalis varies widely in adults ranging usually from 3 to 6 mm [7]. However, there are no diagnostic criteria for "prominent" crista terminalis and its prevalence during TTE examination is unknown. If the prominence of the crista terminalis is superior, then a right atrial mass on TTE can appear when imaged tangentially. The presence of the superior vena cava in the TTE image might suggest that the right atrial mass is a prominent crista terminalis. However, superior vena cava is not easily visualized with TTE and in case of uncertainty TEE can be used to differentiate nonpathologic right atrial structures from pathologic ones. Interrogation of the right atrium in the TEE longitudinal plane (bicaval view) allows visualization of the superior and inferior part of the crista terminalis. Once a stable image of this view is obtained, careful clockwise rotation of the probe allows detailed scanning of the posterolateral wall of the right atrium including a comprehensive delineation of the crista terminalis.

There are a few case reports of prominent crista terminalis in the literature, evaluated with different diagnostic tools, and interestingly all of them are female (see Table 1). Although some atrial arrhythmias have been linked to the anatomic architecture of specific structures such as the crista terminalis [8], in all these reports the clinical history was non-specific, and palpitations were present in only one case.

**Table 1:** Case reports on prominent crista terminalis

**Author[Ref]**	**Sex/Age**	**History**	**TTE**	**3D-TTE**	**TEE**	**MRI**
D'Amato [1]	F/71	HTN, AF	X		X	
McKay [2]	F/49	Healthy	X	X		
Akcay [3]	F/51	Dyspnea, palpitations	X		X	
Gaudio [4]	F/68	HTN	X			X
Pharr [5]	F/77	COPD, dyspnea	X		X	
Pharr [5]	F/74	Legs edema	X		X	
Pharr [6]	F/58	Dyspnea	X		X	
Present study	F/26	ESRD	X		X	X

AF = atrial fibrillation; COPD = chronic obstructive pulmonary disease; ESRD = end stage renal disease; HTN = hypertension.

## Conclusions

The crista terminalis is an example of a pseudomass that can be mistaken for a right atrial lesion. An understanding of the anatomy and proper identification of physiological structures in the right atrium on TTE and TEE can avoid misdiagnosis and unnecessary additional tests.

## Consent statement

Written informed consent was obtained from the patient for publication of this case report and accompanying images. A copy of the written consent is available for review by the Editor-in-Chief of this journal.

## Competing interests

The authors declare that they have no competing interests.

## Authors' contributions

AlS performed the transesophageal echocardiogram, reviewed the echocardiographic and MR studies, and wrote the manuscript. SB reviewed and interpreted the echocardiographic studies and has been involved in drafting the manuscript. AmS compared the echocardiographic images to those obtained at cardiac MR and reviewed the literature. PL performed and interpreted the cardiac MR study. WAAM reviewed critically the manuscript. All authors read the final manuscript.

## Supplementary Material

Additional file 1 **Video 1**. Transthoracic echocardiogram, foreshortened 4-chamber view, the right chamber are displayed at the right. An echogenic structure in the upper part of the right atrium is visible. Click here for file

Additional file 2 **Video 2**. Transthoracic echocardiogram, short-axis view at the level of the aorta focused on the right chambers. The structure in the right atrium is sessile and follows the movements of the heart. Click here for file

Additional file 3 **Video 3**. Transesophageal echocardiogram, short-axis view at the level of the aorta. Click here for file

Additional file 4 **Video 4**. Transesophageal echocardiogram, long-axis view at the level of the right atrium (bicaval view). Clockwise rotation of the probe allows the visualization of the structure. This is a ridge extending from the opening of the superior vena cava to the lateral side of the entrance of the inferior vena cava, consistent with a crista terminalis. Click here for file
